# The Color of Drinking Survey Questionnaire for Measuring the Secondhand Impacts of High-Risk Drinking in College Settings: Validation Study

**DOI:** 10.2196/64720

**Published:** 2025-04-07

**Authors:** Agustina Marconi, Reonda Washington, Amanda Jovaag, Courtney Blomme, Ashley Knobeloch, Vilma Irazola, Carolina Muros Cortés, Laura Gutierrez, Natalia Elorriaga

**Affiliations:** 1 UW-Madison, 333 East Campus Mall 8th floor, Madison, WI, 53711, United States, 1 6086928591; 2 Instituto de Efectividad Clinica y Sanitaria (IECS), Dr. Emilio Ravignani 2024, Buenos Aires, Argentina

**Keywords:** validation study, alcohol drinking in college, microaggression, university, student, young adult, undergraduate, survey, questionnaire, reliability, consistency

## Abstract

**Background:**

The “Color of Drinking” is a study conducted at the University of Wisconsin-Madison. It examines the secondhand harms of high-risk drinking on college students of color and explores the connection between alcohol use and the campus racial climate. Since its findings were released in 2018, this study has received significant attention from other college settings around the country.

**Objective:**

This study aims to describe the development of the most recent version of the Color of Drinking questionnaire and to assess its internal consistency, test-retest reliability, and construct validity in a sample of undergraduate students attending the University of Wisconsin-Madison.

**Methods:**

This is an observational, analytic study. Questionnaire design experts revised the original instrument, and in-depth cognitive interviews with students were conducted to evaluate comprehensibility and acceptability. The revised questionnaire was administered 2 times, 3 to 4 weeks apart, in a sample of undergraduate students. The following properties were studied: internal consistency in 4 sets of items (Cronbach α), test-retest reliability among closed-ended questions (κ statistics and intraclass correlation coefficient), and construct validity (associations with other validated instruments, such as the Alcohol Use Disorders Identification Test). For a section of questions showing low reliability, the answers to open questions and other in-depth interviews were carried out, and online surveys were conducted with another sample of undergraduate students to evaluate reliability after changes.

**Results:**

Eight students participated in the in-depth interviews, 177 responses from the online survey were included for the analysis of internal consistency, 115 for test-retest reliability, and 98 for construct validity. The 4 sets of items (sections) evaluated (“impact of alcohol consumption on academics,” “impact of microaggressions,” “witnessing microaggressions and alcohol intoxication,” and “bystanders’ interventions on alcohol intoxication”) presented good internal consistency (Cronbach α between 0.723 and 0.898). Most items showed moderate to substantial test-retest reliability; agreement was from 68.1% to 95.2%, and κ coefficients ranged from 0.214 to 0.8. For construct validity, correlations between the number of drinking days, the maximum number of drinks in a day and the Alcohol Use Disorders Identification Test score were moderate to high, r=0.630 (95% CI 0.533-0.719) and r=0.647 (95% CI 0.548-0.741), respectively. Due to low reliability, a section regarding “health impacts” has been redesigned, including 8 items for the personal consumption of alcohol and the consumption of others (Cronbach α 0.735 and 0.855, respectively; agreement between the first and the second time the questionnaire was administered were 83.4% and 99.1%, and most of the items with κ coefficient from 0.476 to 0.877).

**Conclusions:**

The revised version of the Color of Drinking questionnaire showed acceptable to adequate reliability and construct validity.

## Introduction

The association between alcohol consumption and being a college student is broadly described in the literature. Students often understand drinking during college as part of their higher education experience and a way to socialize with peers [1]. The first years of college involve major transitions, including changes in family bonds, living arrangements, and peer socialization. That type of transition is often associated with risky behaviors, including high levels of alcohol consumption [2,3]. Environmental factors, such as the campus culture, can further [2,3] reinforce this behavior and contribute to its health consequences [4]. However, few studies have examined the potential impact of a high-risk drinking culture on the mental health and well-being of marginalized college students [5] The Color of Drinking study examines differences in narratives and perspectives related to alcohol culture, high-risk drinking, and the racial climate for students of color and White students at an American predominately White institution [6].

Results from the exploratory Color of Drinking Study (2017/2018) revealed key insights into the intersection of alcohol culture and student experiences. Safety concerns in high-risk drinking environments had a greater impact on students of color compared to their White peers. High-risk drinking was also positively associated with students’ sense of connection and belonging. Students of color reported higher rates of abstaining from or avoiding alcohol than White students (15.3% vs 8.4%). Additionally, African American or Black students considered leaving the university at twice the rate of White students (43.6% vs 20.9%), and students of color were more likely to report financial struggles (14.5% vs 7%). White, problematic drinkers scored higher on the Diener Flourishing Scale than students of color (27.3% vs 10.2%). Campus alcohol culture also influenced academic routines, with 46% of students reporting the need to find alternative study spaces and 40% scheduling group meetings around alcohol consumption [6]. Furthermore, African American or Black was the racial group that most frequently witnessed (82.9%) and experienced microaggressions (79.8%). The likelihood of witnessing microaggressions increased with the year in school, from 49.3% among first-year students to 75.8% among fourth-year students. Among students of color, alcohol use in the last 30 days, feeling impacted by other’s consumption of alcohol, and avoiding certain areas due to alcohol consumption were all significantly associated with experiencing microaggressions among students of color [7].

Since the release of its findings, the Color of Drinking has received significant attention from other college settings, media coverage, and numerous requests to replicate this study around the country [8]. As the instrument gained greater prominence as a research tool and a way for campuses to understand their student experiences, the decision was made to conduct a validation study of the Color of Drinking instrument. This validation effort enhances the University of Wisconsin-Madison’s (UW-Madison’s) ability to interpret survey results accurately and provides other institutions with a clearer understanding of the insights they can gain from administering the survey.

Thus, this study aims to describe the development of the last version of the Color of Drinking questionnaire and to evaluate its reliability and validity in a sample of undergraduate students attending UW-Madison.

## Methods

### Study Design

This observational, analytic study incorporated both qualitative and quantitative research components. Experts in questionnaire design reviewed the original version of the questionnaire and in-depth cognitive interviews were conducted with students to evaluate the comprehensibility and acceptability of the instrument. The revised version of the questionnaire was administered to a sample of undergraduate students at UW-Madison twice, with an interval of 3 to 4 weeks. This timeframe was selected to reduce the likelihood of participants remembering their previous responses while avoiding a gap long enough to span different semesters, which could impact the comparability of recall. Other validated instruments were administered alongside the first administration of the Color of Drinking questionnaire: (1) the Alcohol Use Disorders Identification Test (AUDIT), the microaggressions subscale from the Racial and Ethnic Microaggression Scale (REMS) [9], and the Sense of Belonging Index (SBI). The following properties were evaluated: internal consistency, test-retest reliability, and construct validity.

### Participants

The study was conducted in a sample of undergraduate students aged ≥18 years at UW-Madison.For in-depth cognitive interviews, an opportunistic sample of students (n=8), ensuring variability in age, gender, and ethnicity or race among students with freshman, sophomore, junior, and senior standing, was selected. For the online survey the minimum sample size was estimated in 100 participants. This sample size was determined to assess the construct validity by applying a 2-sample *t* test, based on data previously published from the REMS subscale in a sample of undergraduate students, considering a mean difference of 0.5, an SD of 0.89, an error α of .05 and a statistical power (1-β) of .80 [10]. This sample size is also adequate for assessing test-retest reliability (n=63 participants completing the survey on 2 occasions), based on an anticipated intraclass correlation of 0.7 (with a minimum acceptably threshold of 0.5 [11]), a significance level (α) of .05 and β of .20 [12,13].

Student identity data was obtained from the UW-Madison Office of the Registrar and sampled self-identified White and censused self-identified undergraduate students of color or international students. Based on the recruitment rate from the study previously conducted in the same population [7], a random sample of 300 self-identified White undergraduate students and 200 self-identified undergraduate students of color or international students were invited to participate. Each student received an email with the invitation to participate, and 2 reminder emails were sent. It was explained that there were 2 opportunities to be part of the validation of the Color of Drinking Survey (a qualitative Zoom [Zoom Communications, Inc] interview or an online survey administered twice). Participation was voluntary, and invitations were sent via listservs. Thus, students had the option not to respond to the task. Participants who had completed the first questionnaire were contacted by email with the invitation to complete the second questionnaire.

### Color of Drinking Questionnaire

The original instrument was developed by the University Health Services at the UW-Madison [6]. The dimensions of interest were selected, and a list of questions was developed by an interdisciplinary team composed of a Subject Matter Expert on Alcohol (RW) and a Physician, Epidemiologist specialist in Public Health (AM), based on information gathered during interviews with students and a framework based on previous research [5]. The instrument explored the following dimensions: (1) personal alcohol consumption, (2) academics and alcohol drinking culture, (3) locations (avoiding areas at campus due to concerns of the alcohol use of others or where feeling unsafe), (4) the impact of others’ alcohol use, (5) experiencing microaggressions (targeting students of color only), (6) bystander witnessing and intervention in situations of microaggressions or alcohol intoxications), (7) impact on personal health and well-being, and (8) overall experience and sense of belonging at the campus. The tool also included questions about sociodemographic characteristics such as age, gender, race or ethnicity, and financial situation. The questionnaire included both open-ended and closed-ended questions. Questions about experiencing microaggressions were asked only to students of color, under the definition of microaggressions used by the authors: “racial microaggressions are an everyday manifestation of oppression that brings psychological consequences to target groups” [14]. The well-being section included a validated scale, the Diener Flourishing Scale, consisting of 8 items [15]. The questionnaire was previously administered in 2015, 2017, and 2018 to more than 1100 students; thus, the research team has previously analyzed useful information from both closed-ended and open-ended questions.

During the first phase of the validation study, the original version was revised by a group of researchers with expertise in questionnaire design, obtaining a preliminary draft version. Later, a series of cognitive interviews were conducted with a sample of students. They consisted of the flexible administration of the draft questionnaire and relied heavily on verbal probing to improve the instrument [16,17]. An “inspect-and-repair” model was used to inspect the items, detect flaws, and repair them. If the interviewer identified any problem with 1 or more questions (about comprehension, recall, difficulty to answer, response, or other), they would rephrase the items to test a potential solution [18]. The interviews were focused on the questions developed for the questionnaire and did not include the already validated Diener Flourishing Scale or the section on sociodemographic characteristics. After the series of interviews, a new version of the questionnaire was produced.

### Assessment of the Psychometric Properties

#### Internal Consistency

This property is a measure of the intercorrelation among items and hence the consistency in the measurement of the intended construct [19]. The internal consistency was evaluated in the following sets of items: (1) impact of alcohol consumption on academics (Q8. How often have you experienced the following during the current semester? items: 8.1 to 8.5), (2) impact of microaggressions (Q17. How much did the microaggressions impact your… items: 17.1 to 17.3?), (3) witnessed microaggressions (Q25. Have you witnessed any of the following? ...items: 25.1 to 25.8), and (4) bystander intervention (Q26. Would you intervene in the following situations? ...items: 26.1 to 26.4)

#### Test-Retest Reliability

Test-retest reliability, a measurement of reproducibility, was evaluated for closed-ended questions.

#### Construct Validity

To assess construct validity, participants also completed 3 validated scales: the AUDIT [20], the SBI [21], and the “Workplace and School Microaggressions” Scale from the REMS [9]. They also reported their Grade Point Average (GPA).

The AUDIT is a 10-item questionnaire with a range of possible scores from 0 to 40. Scores ≥7 and 8 have been proposed as “hazardous and harmful alcohol use” among women and men, respectively [20,22]. It was expected that (1) the median number of drinking days and the maximum number of drinks in a day during the last 2 weeks measured by the Color of Drinking questionnaire are greater among those scoring ≥7 than those scoring <7 in the AUDIT instrument, and (2) the number of drinking days and the maximum number of drinks per day during the last 2 weeks are correlated with the AUDIT score.

The validated “Workplace and School Microaggressions” subscale from the REMS, included 5 items describing experienced microaggressions in the last 6 months, with possible score ranges from 0 to 5. It was expected that those who answered that they had experienced a microaggression on campus or surrounding areas (Color of Drinking, Q14) score higher in the REMS subscale than those who did not have such experience [9].

The SBI is a validated 6-item questionnaire with possible scores ranging from 5 to 30 (the greater the score, the more the sense of belonging). It was hypothesized that the SBI score is lower among those who reported that the alcohol culture had impacted their overall sense of belonging at UW-Madison in comparison to those who reported that the alcohol culture had not affected it [21].

Finally, the academic performance was operationalized as self-reported accumulated and last-term GPA. It was expected that academic performance was better among those students who responded that they never received a poor grade because they had chosen to drink instead of study during the semester.

### Redesign and Assessment for Questions With Low Performance and Developing the Final Questionnaire

For a section of questions showing low reliability, the answers to the open-ended questions and other series of in-depth interviews were used to generate a revised set of questions. Then, online surveys were conducted with another sample of undergraduate students to evaluate reliability after changes.

### Data Analysis

Descriptive statistics were calculated for the population and each item. Hypothesis tests were selected according to the variable distribution. Wilcoxon rank sum test with continuity correction and Kendall correlation coefficients were used to test the hypotheses related to construct validity.

The internal consistency was evaluated by calculating the Cronbach α coefficient by dimension. Additionally, we calculated the variation in the α coefficient after excluding each item from the analysis to assess the effect of dropping a particular item. A Cronbach α value of at least 0.7 is usually interpreted as adequate internal consistency [23]. Low values of α represent poor interrelatedness between items.

To assess test-retest reliability, the Cohen κ coefficient (dichotomous data), the Cohen weighted κ coefficient (ordinal data), and the intraclass correlation coefficient (for continuous data) and their 95% CI were calculated. The higher the coefficient, the stronger the test-retest reliability [13]. κ values between 0.41 and 0.6 were considered to be of moderate reliability, between 0.61 and 0.8 as substantial reliability and between 0.81 and 1 very high reliability [19]. κ is influenced by the trait prevalence and basal rates. κ may be low even though there are high levels of agreement and even though individual ratings are accurate, thus we have also calculated the proportion of observed and expected agreement only by chance. For the interpretation of the intraclass correlation coefficient, values lower than 0.5 were considered indicative of poor reliability, values between 0.5 and 0.75 indicate moderate reliability, values between 0.75 and 0.9 good reliability, and values higher than 0.9 excellent reliability [11].

All the analyses considered α <.05 as statistically significant. Analyses were performed using R Statistical Software (version 4.1.3; R Foundation) and RStudio statistical software (version 2022.02.3 for Windows).

### Ethical Considerations

This study was approved by the Institutional Review Board of the University of Wisconsin (Protocol Institutional Review Board ID 2017‐0930). This study was conducted according to the guidelines established in the Declaration of Helsinki for studies involving human subjects. Written informed consent was obtained from all participants in the in-depth interviews, and online consent before the access to the survey in the Qualtrics platform. Students who completed the qualitative interview received US $20 on their campus card. Participants who completed both sets of surveys received US $10 on their student campus card. Data was de-identified for anaylsis.

## Results

### Cognitive Interviews

Table S1 in Multimedia Appendix 1 presents the characteristics of the students who participated in the interviews. The sample included 8 students, aged 19 to 22 years, representing diverse ethnicities, genders, and undergraduate years. The interview duration ranged from 20 minutes to 1 hour.

Overall, participants found most questions easy to answer and acceptable, with their interpretations “in their own words” aligning with the intended purpose of each question. However, for a few questions, 1 or more participants suggested that the interpretation could be improved by incorporating definitions, clarifications, or more specific terminology. Revisions based on these interviews are detailed in Table S2 in Multimedia Appendix 1.

The first version to assess the psychometric properties consists of 31 items (19 core questions+12 skip questions). Additionally, the survey includes 12 questions about sociodemographic characteristics.

### Assessment of Psychometric Properties

A total of 181 students completed the first administration of the questionnaire between June and November 2022. Of these, 116 also completed the second administration. Six observations were excluded from the analysis due to missing answers in >50% of the questions (4 from the first administration and 2 from the second administration). All of them were from the group of students of color. Thus, 177 responses were available for the analysis of internal consistency, 115 for test-retest reliability assessment and 98 for construct validity.

Participants’ characteristics for each substudy are shown below in Table 1. Students of color represented 48.6% (86/177), 43.5% (50/115), and 57.1% (56/98) in substudies of assessment of internal consistency, test-retest assessment, and construct validity, respectively. Most of the students of color were Asian or Asian American, followed by those of Hispanic, Latinx, or Spanish origin. There were students representing all stages of academic studies. A larger number of students lived off campus, and the most frequent current financial situation was described as “tight but doing fine.”

**Table 1.** Demographic characteristics of the participants, the Color of Drinking validation study, University of Wisconsin-Madison, 2022.

Characteristics	Internal consistency (n 177)	Test-retest (n 115)	Construct (n 98)
Ethnicity^a^			
American Indian or Alaskan Native	3	3	3
Asian or Asian American	75	45	50
Black or African American	7	5	6
Hispanic, Latinx, or Spanish	14	7	11
Middle Eastern or North African	2	1	1
White	91	65	42
Biracial	3	2	3
Multiracial	1	2	1
Other identity	7	2	3
Gender			
Female	118	86	62
Male	50	23	30
Gender diverse	9	6	6
Self-identification			
Disabled person	34	21	22
Able-bodied or not disabled	117	79	66
Prefer not to answer	26	15	10
Stage of academic studies			
1st year undergraduate	20	8	17
2nd year undergraduate	42	25	30
3rd year undergraduate	48	30	20
4th year undergraduate	42	36	27
5th year undergraduate or other	16	15	4
Prefer not to answer	9	1	0
Living arrangement			
On campus	43	21	31
Off campus	122	92	66
Other	2	0	1
Prefer not to answer	10	2	0
Current financial situation			
It’s a financial struggle	22	13	10
It’s tight but I’m doing fine	80	53	54
Finances aren’t really a problem	65	47	34
Prefer not to answer	10	2	0
College or school			
College of Agricultural and Life Sciences (CALS)	28	21	14
College of Engineering	21	14	9
College of Letters and Science (L&S)	89	59	56
Wisconsin School of Business	16	11	11
School of Education	13	9	7
School of Human Ecology	4	3	1
School of Nursing	5	4	4
School of Pharmacy	4	3	2
Nelson Institute for Environmental Studies	3	2	1
School of Veterinary Medicine	1	1	0
School or college not listed	4	3	1
Prefer not to answer	9	2	0

a Multiple answer options were allowed.

### Internal Consistency

The four dimensions evaluated presented good internal consistency, with Cronbach α coefficients ranging from 0.723 to 0.898 (Table 2).

**Table 2.** Internal consistency of 4 sets of items: impact of alcohol consumption on academics, the impact of microaggressions, witnessing microaggressions and alcohol intoxication, and bystander interventions on alcohol intoxication: Color of Drinking validation study, UW-Madison^a^, 2022.

Dimension, Q^b^, and items	Cronbach α coefficient (95% CI)	Cronbach α coefficient if an item is dropped
Impact of alcohol consumption on academics (n=177)	0.795 (0.689-0.854)	
Q8 How often have you experienced the following during the current semester?^c^		
8.1 I have been too hungover to attend class		0.74
8.2 I chose to drink instead of study		0.77
8.3 I received a poor grade because I chose to drink instead of study		0.78
8.4 Missed a class because of alcohol use		0.74
8.5 Performed poorly on an assignment due to alcohol use		0.74
Impact of microaggressions (n=22)	0.898 (0.832-0.94)	
Q17. How much did the microaggressions impact your …?^d^		
17.1 Sense of belonging at UW-Madison?		0.88
17.2 Health and wellbeing?		0.83
17.3 Ability to learn at UW-Madison?		0.85
Witnessing microaggressions and alcohol intoxication (n=12)	0.723 (0.643-0.782)	
Q25. Have you witnessed any of the following?^e^		
25.1 A friend makes a discriminatory statement to a stranger		0.72
25.2 A stranger makes a discriminatory comment to a friend		0.68
25.3 Your alcohol-intoxicated friend makes a discriminatory comment to a stranger		0.7
25.4 An alcohol-intoxicated stranger makes a discriminatory comment to a friend		0.68
25.5 A friend is passed out in a public space and unconscious due to alcohol		0.72
25.6 A stranger is passed out in a public space and unconscious due to alcohol		0.67
25.7 A friend being transported to detox because of alcohol intoxication		0.7
25.8 A stranger being transported to detox because of alcohol intoxication		0.7
Bystanders’ interventions (n 112)	0.724 (0.649-0.779)	
Q26. Would you intervene in the following situations?^e^		
26.1 A friend is passed out in a public space and unconscious due to alcohol		0.67
26.2 A stranger is passed out in a public space and unconscious due to alcohol		0.68
26.3 A friend being transported to detox because of alcohol intoxication		0.65
26.4 A stranger being transported to detox because of alcohol intoxication		0.64

a UW-Madison: University of Wisconsin-Madison.

b Q: question.

c Answer categories: never, rarely, sometimes, often, and most of the time

d Answer categories: not at all, slightly, somewhat, quite a bit, and a great deal.

e Answer categories: yes and no.

### Test-Retest Reliability

Approximately 33% (38/115) and 30% (35/115) of the students reported that they do not drink alcohol or did not drink during the last 30 days in test and retest surveys, respectively. The mean number of drinking days within the last 2 weeks was 1.4 and 1.8 for test and retest, respectively. In summary, the section “alcohol use” showed moderate to substantial reliability (Table S3 in Multimedia Appendix 1).

On test and retest surveys, 9.6% (11/114) of the participants reported that the alcohol culture at UW-Madison had impacted their academics (Table S4 in Multimedia Appendix 1). Most items on the section “alcohol culture and academics” showed moderate to substantial test-retest reliability, but 1 of them showed low test-retest reliability.

On test and retest surveys, 51.8% (59/114) of the participants reported that during the semester they had avoided specific areas on or off campus due to concerns about alcohol use by others. The proportion in which participants were negatively impacted by other students’ alcohol consumption was 35% (40/113) for the test and 28% (32/113) for the retest. Items from these 2 sections, “areas avoided” and “impact of by other students’ alcohol consumption” also showed mostly moderate to substantial test-retest reliability (Table S2 in Multimedia Appendix 1).

Among the students of color, 47% (29/62) and 40% (25/62) reported having experienced a microaggression on campus or surrounding areas on test and retest surveys. On test and retest surveys, 71% (81/114) and 73% (83/114) of the students had witnessed at least once a microaggression on campus or surrounding areas during their time at UW-Madison. A summary of test-retest reliability assessment for experiencing, witnessing, and bystander intervention of microaggressions and alcohol intoxications is presented in Table S4 in Multimedia Appendix 1. Most of the items from these dimensions showed moderate to substantial test-retest reliability.

From test and retest surveys, 33% (37/112) and 25% (28/112) of the participants answered that their own or alcohol consumption by other students impacted their health during the time they attended UW-Madison. At test and retest, 43% (49/113) and 42% (47/113) of the students reported that the alcohol culture had impacted their overall sense of belonging at UW-Madison. Most of the items from the section “impact on health and sense of belonging” showed moderate to substantial test-retest reliability, except for 1 related to the impact on health (Table S4 in Multimedia Appendix 1).

### Construct Validity

Students with AUDIT scores ≥7 reported a higher number of drinking days within the last 2 weeks than those with lower AUDIT scores. A similar association was found regarding the maximum number of drinks in a day and the AUDIT score with a cutoff point of 7 (Table 3). Correlations between the number of drinking days, the maximum number of drinks in a day and the AUDIT score as a continuous variable were moderate to high, *r*=0.630 (95% CI 0.533-0.719) and *r*=0.647 (95% CI 0.548-0.741), respectively (Table 3).

A few students reported that they received a poor grade because they had chosen to drink instead of study. Their GPA was lower than the other students’ (Table 4).

**Table 3.** Construct validity. Drinking days within the last 2 weeks and a maximum number of drinks in a day among participants with AUDIT^a^ scores less than and greater than or equal to 7. CoD^b^ validation study, University of Wisconsin-Madison, 2022.

	AUDIT score		
	<7 (n=62)	≥7 (n=35)	
Variables from the Color of Drinking questionnaire	Median(IQR)	Median (IQR)	*P* value^c^
Drinking days within the last 2 wk	0 (0‐2)	3 (0‐5)	<.0001
Maximum number of drinks in a day	0 (0‐3)	6 (3‐10)	<.0001

a AUDIT: Alcohol Use Disorders Identification Test.

b CoD: Color of Drinking.

c Wilcoxon rank sum test.

**Table 4.** Construct validity. Self-reported grade point average (GPA^a^), microaggression subscale from the REMS^b^ and SBI^c^ according to selected items from the CoD^d^ questionnaire validation study. UW-Madison^e^, 2022.

Dimension: CoD questionnaire item		Variable (other instrument)	N	Mean (SD)	Median (IQR)	*P* value^f^
Dimension: alcohol consumption and academics						
	CoD questionnaire, item 8.3 (“I received a poor grade because I chose to drink instead of study”)	GPA, accumulated				.04
	At least once during the current semester		7	3.19 (0.57)	3.3 (2.7‐3.68)	
	Never		71	3.6 (0.37)	3.7 (3.4‐3.9)	
		GPA, last term				.02
	At least once during the current semester		6	2.9 (0.77)	2.9 (2.28‐3.55)	
	Never		70	3.59 (0.58)	3.8 (3.5‐4)	
Dimension: microaggressions						
	CoD questionnaire, Q13. “In your time at UW-Madison, have you experienced a microaggression on campus or surrounding areas?”	Microaggressions subscale from REMS^g^				<.001
	Yes		27	2.04 (1.83)	2 (0‐3.5)	
	No		40	0.68 (1.4)	0 (0‐1)	
Dimension: impact on the sense of belonging						
	CoD questionnaire, Q28. “Has the alcohol culture impacted your overall sense of belonging at UW-Madison?”	SBI^h^				<.001
	Yes		41	16.07 (2.23)	16 (15-18)	
	No		57	18.04 (2.15)	18 (17‐19)	

a GPA: grade point average.

b REMS: Racial and Ethnic Microaggression Scale.

c SBI: Sense of Belonging Index.

d CoD: Color of Drinking.

e UW-Madison: University of Wisconsin-Madison.

f Wilcoxon rank sum test (*P* value).

g Score ranges from 0 to 5.

h Score ranges from 5 to 30.

Additionally, 27 of 67 students of color answered that in their time at UW-Madison they had experienced a microaggression on campus or surrounding areas. They scored higher on the REMS than the rest of the students.

Lastly, 41 of 98 students answered that the alcohol culture impacted their overall sense of belonging at UW-Madison. These students scored lower in the SBI in comparison with the rest of the students (Table 4).

### Redesign and Assessment for Questions With Low Performance and Developing the Final Questionnaire

The “health impacts” section showed a low test-retest reliability. When the qualitative responses were evaluated, students brought up different interpretations but also very profound answers and concepts, such as mental health struggles or experiencing sexual assault. Based on these results, the decision was made to rewrite and retest the validity of this question grounded on the evaluation of content validity, and other series of in-depth interviews were used to generate a revised set of questions. Eight items were developed for the personal consumption of alcohol on diverse aspects of health, and the same number of items were included for the consumption of others.

Online surveys were conducted including the 16 new items with another sample of undergraduate students selected following the same procedures. The sample included 118 students who completed the first questionnaire. Most participants were women (84, 71%), and 66 participants were self-identified as White. Sixty-seven participants also completed the second questionnaire.

The results for internal consistency and test-retest reliability were Cronbach α values of .735 and .855, for each set of questions, respectively, and most of the items presented κ coefficients from 0.476 to 0.877 (Tables S5 and S6 in Multimedia Appendix 1).

## Discussion

The validation process outlined here included the assessment of reliability and validity of the Color of Drinking questionnaire, used in an exploratory study of the impact of UW-M’s alcohol culture on students of color. The process of validating the original questionnaire resulted in a revised instrument, that showed good internal consistency, acceptable to good test retest reliability for most items and good construct validity.

In the alcohol and academics dimension, we found that questions related to personal academic consequences had higher internal consistency and a higher Cronbach α than the other questions in the same dimension. Questions about the secondhand negative academic consequences were found to be highly relevant but not highly correlated with each other. The question “my professors or TAs invited me out to places where alcohol is served” tested poorly when placed with the other secondhand academic consequences. For the finalized questionnaire we decided to separate the personal academic consequences, and secondhand negative consequences as 2 separate subdomains. We also removed the aforementioned question due to some unclearness about the scope and meaning of the item identified during the qualitative interview and little consistency with other items in the expected subdomain.

In the bystander intervention dimension, there were issues with test-retest reliability among 4 questions. Bystander discrimination questions from the questionnaire had low test-retest reliability. The questions were more prone to variation over time than the other bystander questions. The decision was made to remove these 4 questions from the finalized version of the questionnaire. Other validation processes in college settings show adequate validity and reliability when assessing other types of bystander interventions [24].

The health impacts section showed a low test-retest reliability, however based on responses there was important information to include in this section. Thus, the section was rewritten and tested, reaching good reliability.

A questionnaire designed for populational health studies should capture what it was meant to collect when designed in a reliable manner [25,26]. This validation process will not only allow us to measure what we want to measure but also will make our data comparable over time and with other college settings [27] .

This study has limitations. Although there was a random selection of students invited to participate, we recognize that those more sensitive to racial issues may have been more likely to take part in this study. This potential selection bias could influence the generalizability of our findings to the broader university population.

In conclusion, the process of validating the original questionnaire for the Color of Drinking study resulted in a revised instrument, that showed good internal consistency, acceptable to good test-retest reliability for most items and good construct validity.

## Supplementary material

10.2196/64720 Multimedia Appendix 1 Additional materials: additional tables S1-S6.

## Abbreviations

AUDIT: Alcohol Use Disorders Identification Test
REMS: Racial and Ethnic Microaggression Scale
SBI: Sense of Belonging Index
UW-Madison: University of Wisconsin-Madison
