# Supplementary material for: The Color of Drinking Survey Questionnaire for Measuring the Secondhand Impacts of High-Risk Drinking in College Settings: Validation Study
Source: Interact J Med Res. 2025 Apr 7;14:e64720. doi: 10.2196/64720 (PMC11996142; doi:10.2196/64720)
Supplement: Multimedia Appendix 1 [file ijmr-v14-e64720-s001.docx]

**Table S1.** Color of Drinking validation study. Characteristics of the students who had participated in cognitive interviews (n=8).

| **Respondent ID** | **Gender** | **Ethnicity/ Race** | **Age** | **Undergraduate year** |
| --- | --- | --- | --- | --- |
|  |  |  | (years) |  |
|  |  |  |  |  |
| 1 | Female | White | 19 | (1) Freshman |
| 2 | Male | Color | 20 | (2) Sophomore |
| 3 | Female | Color | 22 | (4) Senior |
| 4 | Male | White | 21 | (3) Junior |
| 5 | Female | International | 21 | (4) Senior |
| 6 | Other identity | White | 22 | (4) Senior |
| 7 | Female | International | 19 | (2) Sophomore |
| 8 | Female | International | 22 | (3) Junior |

**Table S2.** Color of Drinking validation study. Changes in the questionnaire based on the analysis of the in-depth interviews.

| **Section** | **Original question** | **Changes** |
| --- | --- | --- |
|  |  |  |
| Alcohol consumption | *Within the last 30 days, how many days did you use alcohol (beer, wine, malt beverage, alcopop liquor)?* | *“use alcohol” was replaced with “drink alcohol”*  *Within the last 30 days, how many days did you drink alcohol (beer, wine, malt beverage, alcopop liquor)?* |
| Alcohol culture and academics | *How often have you experienced the following during the current semester?... (Never, rarely, sometimes, often, always)* | -“always” was replaced with  “most of the time”  -“not applicable” was added as an option |
| Avoinding areas and feeling unsafe | *….*  *Where on the UW-Madison Campus do you feel safe?* | The question was split into two, clarifying the time of day as follows:  *a. Where on the UW-Madison Campus do you feel safe during the day?*  *b Where on the UW-Madison Campus do you feel safe during the night?* |
| Microaggressions | *For this study, microaggressions were defined as “brief and commonplace daily verbal, behavioral, or environmental indignities, whether intentional or unintentional, that communicate hostile, derogatory, or negative racial slights and insults towards people of color (Sue et al, 2007).*  *In your time at UW-Madison, have you experienced a microaggression on campus or surrounding areas?  · Yes  · No* | *Two sentences were inserted in the introduction to the question:*  *Now, we will ask you about microaggressions related or not related to alcohol consumption.*  *For this study, microaggressions were defined as “brief and commonplace daily verbal, behavioral, or environmental indignities, whether intentional or unintentional, that communicate hostile, derogatory, or negative racial slights and insults towards people of color (Sue et al, 2007). People of color are defined as someone who does not identify solely as white* |
| *Microaggressions* | *Who have been the sources of microaggressions you have experienced on campus or surrounding areas this semester (check all that apply)  Classmates  Roommates  Other Peers/Friends  Other Students  Faculty  Staff  Visitors to Campus  Other Adults with unknown affiliation  Parents  Fraternity/Sorority Member*  *Other (please specify) ________* | *For 3 response alternatives clarifications were included:*  *-Roommates (living in the same room, floor or dorm)*  *-Faculty/Advisors*  *-Staff/Other employees on campus* |
| *Microaggressions* | *During this semester, have you experienced any microaggressions from other students on campus when they were intoxicated?* | *…‘when they were intoxicated’ was replaced with ‘when they had been drinking alcohol’* |
| *Health Impact* | *Has alcohol use impacted your health (physical, mental, overall well-being) in the time you have attended UW-Madison?* | *Whose alcohol consumption were added*  *Has your personal alcohol consumption or others’ alcohol consumption impacted your health (physical, mental, overall well-being) in the time you have attended UW-Madison?* |
| *Overall experience and sense of belonging* | *Have you considered leaving the University?*  *Yes*  *No* | *“the University” was replaced with the name of the institution:*  *Have you considered leaving UW-Madison?*  *[(e.g. transferring or dropping out)]* |

**Table S3.** Assessment of test-retest reliability for the section of alcohol consumption, Color of Drinking Study. UW-Madison 2022.

| Question | n | Kappa (95% CI) | ICC (95% CI) |
| --- | --- | --- | --- |
| Q4. Number of days that had drunk alcohol within the last 30 days | 115 | 0.690 (0.600 - 0.781)^a^ | 0.689 (0.579 - 0.774)^b^ |
| Q5. Alcohol consumption during the past two weeks | 78 |  |  |
| Q5.a. Number of drinking days |  |  | 0.537 (0.360 - 0.677) |
| Q5.b Max. number of drinks in a day |  |  | 0.642 (0.492 – 0.756) |

95% CI, 95% confidence interval; ICC, Intra-class correlation coefficient; Kappa (Cohen’s Kappa coefficient)

^a^ Weighted Kappa

^b^ Data were analyzed as continuous, after assigning the midpoint of the category

**Table S4.** Assessment of test-retest reliability for alcohol culture and academics, avoiding areas and feeling unsafe, microaggressions, impact on health and sense of belonging, Color of Drinking Study. UW-Madison 2022.

| Section/ Question | | n | | Test | | Retest | | Kappa (95% CI) | | Agreement | | Expected agreement by chance |  |  |
| --- | --- | --- | --- | --- | --- | --- | --- | --- | --- | --- | --- | --- | --- | --- |
| *Alcohol culture and academics* | |  | |  | |  | |  | |  | |  |  |  |
| Q7. Has the alcohol culture at UW-Madison affected your academics? (Yes)    *Student’s alcohol consumption*  Q8 How often have you experienced the following during the current semester? (At least once)* | | 114 | | 9.6% | | 9.6% | | 0.698 (0.470 - 0.925) | | 94.74% | | 82.56% | | |
| 8.1 I have been too hungover to attend class | | 106 | | 18.9% | | 13.2% | | 0.512 (0.292 - 0.732) | | 86.79% | | 72.91% | | |
| 8.2 I chose to drink instead of study | | 106 | | 31.1% | | 24.5% | | 0.602 (0.434 - 0.770) | | 83.96% | | 59.61% | | |
| 8.3 I received a poor grade because I chose to drink instead of study | | 106 | | 8.5%1 | | 7.5% | | 0.680 (0.417 - 0.943) | | 95.28% | | 85.24% | | |
| 8.4 Missed a class because of alcohol use | | 106 | | 16% | | 9.4% | | 0.453 (0.207 – 0.699) | | 87.74% | | 77.55% | | |
| 8.5 Performed poorly on an assignment due to alcohol use | | 106 | | 13.2% | | 6.6% | | 0.366 (0.092 – 0.639) | | 87.74% | | 80.65% | | |
| *Drinking culture*  How often have you experienced the following during the current semester? (At least once)^a^ | |  | |  | |  | |  | |  | |  | | |
| Q9.1. My professors or TAs brought up the alcohol culture in the classroom, not as part of the academic content of the class | | 106 | | 35.8% | | 35.8% | | 0.630 (0.476 – 0.785) | | 82.08% | | 57.67% | | |
| Q9.2. When assigned group work, group meetings were scheduled around drinking days | | 106 | | %18.1 | | 15.2% | | 0.417 (0.189 – 0.646) | | 83.81% | | 72.18% | | |
| Q9.3. I had to find alternative study spaces because of the drinking culture | | 106 | | 30.6% | | 28.7% | | 0.422 (0.236 – 0.608) | | 75.93% | | 58.28% | | |
| *Avoiding areas and feeling unsafe* | |  | |  | |  | |  | |  | |  | | |
| Q10. During the current semester, did you avoid specific areas on or off campus due to concerns of the alcohol use of others? (Yes) | | 114 | | 51.8% | | 51.8% | | 0.613 (0.468 - 0.758) | | 80.70% | | 50.06% | | |
| Q12a. Is there a place at UW-Madison Campus where you feel unsafe (physically or emotionally) during the day? (Yes) | | 114 | | 7.9% | | 9.6% | | 0.452 (0.167 - 0.737) | | 91.23% | | 83.98% | | |
| Q12b. Is there a place at UW-Madison Campus where you feel unsafe (physically or emotionally) during the night? (Yes) | | 114 | | 64% | | 59.6% | | 0.610 (0.461 - 0.759) | | 81.58% | | 52.71% | | |
| *Impact of other students’ alcohol use* | |  | |  | |  | |  | |  | |  | | |
|  | |  | |  | |  | |  | |  | |  | | |
| Q13. Have you been negatively impacted by other student(s)’ alcohol consumption? (Yes)  *Having experienced a microaggression* | | 113 | | 35.4% | | 28.3% | | 0.675 (0.531 - 0.820) | | 85.84% | | 56.33% | | |
| Q14. In your time at UW-Madison, have you experienced a microaggression on campus or surrounding areas? | | 62 | | 46.8% | | 40.3% | | 0.673 (0.489 – 0.857) | | 83.87% | | 50.62% | | |
| Q17. How much did the microaggressions impact your…b | |  | |  | |  | |  | |  | |  | | |
| 17.1 Sense of belonging at UW-Madison? (Somewhat, quite a bit or a great deal) | | 22 | | 77.3% | | 68.2% | | 0.546 (0.162 – 0.930) | | 81.82% | | 59.92% | | |
| 17.2 Health and wellbeing (Somewhat, quite a bit or a great deal) | | 22 | | 50% | | 36.4% | | 0.545 (0.208 – 0.882) | | 77.27% | | 50.00% | | |
| 17.3 Ability to learn at UW-Madison? (Somewhat, quite a bit or a great deal)    *Witnessing and intervention in a microaggression/ alcohol intoxications* | | 22 | | 36.4% | | 36.4% | | 0.80 (0.500 – 1.000) | | 90.91% | | 53.72% | | |
| Q23. In your time at UW-Madison, how often have you witnessed a microaggression on campus or surrounding areas?^c^ (Once a month or more frequently) | | 114 | | 40.4% | | 36.8% | | 0.540 (0.418 – 0.663)d | | 88.89% | | 75.58% | | |
| Q24. Have you intervened in a situation where you witnessed a microaggression on campus or surrounding areas? (Yes) | | 114 | | 21.1% | | 16.7% | | 0.685 (0.513 – 0.857) | | 90.35% | | 69.30% | | |
| Q25. Have you witnessed any of the following? (Yes) | |  | |  | |  | |  | |  | |  | | |
| 25.1 A friend makes a discriminatory statement to a stranger. | | 112 | | 17.9% | | 12.5% | | 0.448 (0.223 – 0.673) | | 85.71% | | 74.11% | | |
| 25.2 A stranger makes a discriminatory comment to a friend. | | 112 | | 43.8% | | 40.2% | | 0.524 (0.364 – 0.683) | | 76.79% | | 51.23% | | |
| 25.3 Your alcohol-intoxicated friend makes a discriminatory comment to a stranger. | | 112 | | 13.4% | | 9.8% | | 0.566 (0.327 – 0.805) | | 91.07% | | 79.42% | | |
| 25.4 An alcohol-intoxicated stranger makes a discriminatory comment to a friend | | 112 | | 35.7% | | 33.9% | | 0.488 (0.319 – 0.658) | | 76.79% | | 54.59% | | |
| 25.5 A friend is passed out in a public space and unconscious due to alcohol. | | 112 | | 20.5% | | 14.3% | | 0.414 (0.198 – 0.629) | | 83.04% | | 71.05% | | |
| 25.6 A stranger is passed out in a public space and unconscious due to alcohol | | 112 | | 63.4% | | 61.6% | | 0.466 (0.297 – 0.635) | | 75.00% | | 53.11% | | |
| 25.7 A friend being transported to detox because of alcohol intoxication | | 111 | | 17.1% | | 12.6% | | 0.468 (0.240 – 0.695) | | 86.49% | | 74.59% | | |
| 25.8 A stranger being transported to detox because of alcohol intoxication | | 111 | | 27% | | 25.2% | | 0.440 (0.251 – 0.629) | | 78.38% | | 61.38% | | |
| Q26. Would you intervene in the following situations? (Yes) | |  | |  | |  | |  | |  | |  | | |
| 26.1 A friend is passed out in a public space and unconscious due to alcohol. | | 109 | | 83.5% | | 88.1% | | 0.214 (-0.019 - 0.447) | | 80.73% | | 75.50% | | |
| 26.2 A stranger is passed out in a public space and unconscious due to alcohol. | | 106 | | 46.2% | | 49.1% | | 0.603 (0.451 - 0.754) | | 80.19% | | 50.07% | | |
| 26.3 A friend being transported to detox because of alcohol intoxication. | | 108 | | 63.9% | | 67.6% | | 0.507 (0.336 - 0.678) | | 77.78% | | 54.89% | | |
| 26.4 A stranger being transported to detox because of alcohol intoxication.  *Impact on Health and sense of belonging* | | 103 | | 27.2% | | 19.4% | | 0.623 (0.446 - 0.799 ) | | 86.41% | | 63.96% | | |
| Q27. Has your personal alcohol consumption or others’ alcohol consumption impacted your health (physical, mental, overall well-being) in the time you have attended UW-Madison? | | 112 | | 33.0% | | 25.0% | | 0.333 (0.147- 0.519) | | 72.32% | | 58.48% | | |
| Q29. Has the alcohol culture impacted your overall sense of belonging at UW-Madison? (Yes) | | 113 | | 43.4% | | 41.6% | | 0.674 (0.536 – 0.811) | | 84.07% | | 51.12% | | |
| Q30. Has the alcohol consumption impacted your overall experience at UW-Madison? (Yes) | | 113 | | 63.7% | | 61.1% | | 0.530 (0.404 – 0.657) | | 68.14% | | 32.08% | | |
| Q31. Have you considered leaving UW-Madison? (Yes) | | 114 | | 21.1% | | 16.7% | | 0.800 (0.658 – 0.941) | | 93.86% | | 69.30% | | |

95% CI, 95% confidence interval; Kappa (Cohen’s Kappa coefficient); Kw, Weighted Kappa

^a^ Answers were classified into the following two categories: a) Never, b) At least once (Rarely, Sometimes, Often or Most of the time)

^b^ Answers were classified into the following two categories: a) Not at all/ slightly; b) Somewhat/Quite a bit/A great deal

^c^ Answers were classified into the following three categories: a) Never; b) A few times a year or less; b) Once a month or more frequently

^d^ Weighted kappa

**Table S5.** Internal consistency of two sets of items: Impact of personal and others alcohol consumption on health and wellbeing. Color of Drinking Validation Study. UW-Madison, 2023.

| Dimension, question (Q) and items | Cronbach Alpha Coefficient | | Cronbach Alpha Coefficient if an item is dropped |
| --- | --- | --- | --- |
| *Perceived impact of personal alcohol consumption on health and wellbeing (n 84)^a^* | 0.7351 | |  |
| **To what extent has your personal alcohol consumption impacted aspects of your health and wellbeing during the time you have attended UW-Madison, overall?^b^**^1^ |  |  |  |
| 1. Mental and emotional health |  | | 0.6968 |
| 2. Physical health |  | | 0.7194 |
| 3. Interpersonal relationships |  | | 0.7102 |
| 4. Safety |  | | 0.7051 |
| 5. Sexual Health |  | | 0.7088 |
| 6. Social connection and belonging |  | | 0.7576 |
| 7. Spirituality |  | | 0.6730 |
| 8. Overall health and wellbeing |  | | 0.6852 |
| *Perceived impact of alcohol consumption of others on health and wellbeing (n 118)* | 0.8551 | |  |
| **To what extent has the alcohol consumption of others impacted aspects of your health and wellbeing during the time you have attended UW-Madison, overall?** ^b^ |  |  |  |
| 1. Mental and emotional health |  | | 0.8259 |
| 2. Physical health |  | | 0.8383 |
| 3. Interpersonal relationships |  | | 0.8356 |
| 4. Safety |  | | 0.8451 |
| 5. Sexual Health |  | | 0.8301 |
| 6. Social connection and belonging |  | | 0.8491 |
| 7. Spirituality |  | | 0.8467 |
| 8. Overall health and wellbeing |  | | 0.8299 |

^a^ Participants who previously answered that drink alcohol

^b^ Answer categories: Very Negatively, Somewhat Negatively, Not impact overall, Somewhat Positively, very Negatively

**Table S6.** Test retest reliability of two sets of items: Impact of personal and others alcohol consumption on health and wellbeing. Color of Drinking Validation Study. UW-Madison, 2023.

|  | Q | Negatively ^a^ | No impact overall | Positively ^b^ | Agreement | Expected Agreement | Kappa_w_ | SE | p-value |
| --- | --- | --- | --- | --- | --- | --- | --- | --- | --- |
|  |  | n | n | n | % | % |  |  |  |
| To what extent has your personal alcohol consumption impacted aspects of your health and wellbeing during the time you have attended UW-Madison, overall? (n=47)^c^ | | | | | | | | | |
| 1.Mental and emotional health | 1 | 9 | 32 | 6 | 87.66 | 80.62 | 0.3634 | 0.1540 | .0091 |
|  | 2 | 20 | 19 | 8 |  |  |  |  |  |
| 2Physical health | 1 | 17 | 29 | 1 | 94.89 | 87.59 | 0.5886 | 0.1276 | <.0001 |
|  | 2 | 17 | 30 | - |  |  |  |  |  |
| 3.Interpersonal relationships | 1 | 4 | 19 | 24 | 83.40 | 76.46 | 0.2950 | 0.1458 | 0.0215 |
|  | 2 | 6 | 17 | 24 |  |  |  |  |  |
| 4. Safety | 1 | 15 | 30 | 1 | 89.79 | 88.38 | 0.1208 | 0.1299 | 0.1762 |
|  | 2 | 16 | 31 | 1 |  |  |  |  |  |
| 5.Sexual health | 1 | 6 | 38 | 2 | 91.74 | 90.91 | 0.0915 | 0.1420 | 0.2598 |
|  | 2 | 2 | 38 | 6 |  |  |  |  |  |
| 6.Social connection and belonging | 1 | 4 | 14 | 29 | 89.36 | 72.81 | 0.6087 | 0.1406 | <.0001 |
|  | 2 | 4 | 8 | 35 |  |  |  |  |  |
| 7.Spirituality | 1 | 4 | 43 | - | 99.15 | 93.06 | 0.8773 | 0.1541 | <.0001 |
|  | 2 | 6 | 41 | - |  |  |  |  |  |
| 8. Overall health and wellbeing | 1 | 9 | 33 | 5 | 92.34 | 85.39 | 0.4758 | 0.1662 | 0.0021 |
|  | 2 | 13 | 28 | 6 |  |  |  |  |  |
|  |  |  |  |  |  |  |  |  |  |
| To what extent has the alcohol consumption of others impacted aspects of your health and wellbeing during the time you have attended UW-Madison, overall? (n=67) | | | | | | | | | |
| 1.Mental and emotional health | 1 | 34 | 30 | 3 | 86.57 | 78.06 | 0.3878 | 0.1208 | .0007 |
|  | 2 | 27 | 37 | 3 |  |  |  |  |  |
| 2Physical health | 1 | 12 | 55 | - | 95.52 | 88.93 | 0.5956 | 0.1125 | <.0001 |
|  | 2 | 10 | 56 | 1 |  |  |  |  |  |
| 3.Interpersonal relationships | 1 | 28 | 26 | 13 | 85.37 | 66.01 | 0.5697 | 0.1138 | <.0001 |
|  | 2 | 29 | 22 | 16 |  |  |  |  |  |
| 4. Safety | 1 | 30 | 37 | - | 91.94 | 84.07 | 0.4941 | 0.1206 | <.0001 |
|  | 2 | 27 | 39 | 1 |  |  |  |  |  |
| 5.Sexual health | 1 | 13 | 53 | 1 | 90.15 | 87.68 | 0.2004 | 0.1120 | 0.0368 |
|  | 2 | 9 | 55 | 3 |  |  |  |  |  |
| 6.Social connection and belonging | 1 | 26 | 22 | 19 | 84.48 | 57.97 | 0.6307 | 0.1071 | <.0001 |
|  | 2 | 23 | 19 | 25 |  |  |  |  |  |
| 7.Spirituality | 1 | 8 | 59 | - | 94.93 | 88.84 | 0.5451 | 0.1123 | <.0001 |
|  | 2 | 7 | 58 | 2 |  |  |  |  |  |
| 8. Overall health and wellbeing | 1 | 20 | 41 | 6 | 93.73 | 83.08 | 0.6294 | 0.1246 | <.0001 |
|  | 2 | 19 | 44 | 4 |  |  |  |  |  |

Q: Questionnaire

^a^ Answer categories: Very Negatively/Somewhat Negatively

^a^ Answer categories: Very Positively/Somewhat Positively

^c^ Participants who previously answered that drink alcohol
